# Comparison of Different Electroporation Parameters on Transfection Efficiency of Sheep Testicular Cells 

**DOI:** 10.22074/cellj.2016.4571

**Published:** 2016-08-24

**Authors:** Sarah Niakan, Banafsheh Heidari, Ghasem Akbari, Zahra Nikousefat

**Affiliations:** 1 Department of Clinical Science, Science and Research Branch, Islamic Azad University, Tehran, Iran; 2 Cellular and Molecular Research Center, Shahrekord University of Medical Sciences, Shahrekord, Iran; 3 Department of Clinical Science, Faculty of Veterinary Medicine, Razi University, Kermanshah, Iran

**Keywords:** Electroporation, Testicular Cells, Transfection, Sheep, Spermatogonial Stem Cells

## Abstract

**Objective:**

Electroporation can be a highly efficient method for introducing the foreign genetic materials into the targeted cells for transient and/or permanent genetic modification. Considering the application of this technique as a very efficient method for drug, oligonucleotide, antibody and plasmid delivery for clinical applications and production of transgenic animals, the present study aimed to optimize the transfection efficiency of sheep testicular cells including spermatogonial stem cells (SSCs) via electroporation.

**Materials and Methods:**

This study is an experimental research conducted in Biotechnology Research Center (Avicenna Research Institute, Tehran, Iran) from September 2013 to March 2014. Following isolation and propagation of one-month lamb testicular cells (SSCs and somatic testicular cells including; Sertoli, Leydig, and myoid cells), the effect of different electroporation parameters including total voltages (280, 320, and 350 V), burst durations (10, 8, and 5 milliseconds), burst modes (single or double) and addition of dimethyl sulfoxide (DMSO) were evaluated on transfection efficiency, viability rate and mean fluorescent intensity (MFI) of sheep testicular cells.

**Results:**

The most transfection efficiency was obtained in 320 V/8 milliseconds/single burst group in transduction medium with and without DMSO. There was a significantly inverse correlation between transfection efficiency with application of both following parameters: addition of DMSO and double burst. After transfection, the highest and lowest viability rates of testicular cells were demonstrated in 320 V/8 milliseconds with transduction medium without DMSO and 350 V/5 milliseconds in medium containing DMSO. Ad- dition of DMSO to transduction medium in all groups significantly decreased the viability rate. The comparison of gene expression indicated that Sertoli and SSCs had the most fluorescence intensity in 320 V/double burst/DMSO positive. However, myoid and Leydig cells showed the maximum expression in 320 V/single burst and/or 350 V/double burst/ DMSO positive.

**Conclusion:**

We optimized the electroporation method for transfection of sheep testicular cells and recommended the application of 320 V/8 milliseconds/single pulse/DMSO negative for transduction of plasmid vector into these cells. Among testicular cells, the most external gene expression was demonstrated in SSC population.

## Introduction

Testicular tissue is comprised of several types of cells including somatic (Sertoli, Leydig, and peritubular myoid cells) and spermatogonial stem cells (SSCs). Due to their unique properties, SSCs as tissue specific adult stem cells are introduced the perfect target for genetic manipulations (1,2). Recently, germ cell modification and production of transgenic animals through the germ line using transplantation of transfected germ cells became one of the most important applications of SSCs (3). Transfection, entrance of foreign DNA into a recipient eukaryotic cell and subsequently integration into chromosomal DNA, is one of the most valuable tools in biological science (4). The important applications of transfection in biological science are the gene function studies, modulation of gene expression, biochemical mapping, mutational analysis and production of recombinant proteins (5,7). 

Germ line transfection may be the only method to study the integration of foreign vectors into chromosomes at zygotene and pachytene stages of prophase I, which is the only period when endogenous homologous recombination occurs and bivalent complexes form (8). The other advantage of germ line transfection is production of transgenic animals in a shorter time. Following insertion of desired genes into SSCs and transplantation of modified germ cells to immunodeficient recipients, the produced haploid cells and subsequent progeny will carry the transgene (9). Several techniques for delivery of DNA into cells have been established including: i. Chemical transfection, method that rely on carrier molecules for transduction, ii. Physical transfection, method that deliver nucleic acids directly to the cytoplasm, and iii. Viral transfection, method that make use of genetically engineered viruses for cell transduction (6,7,10,14). Among the transfection methods, chemical (with liposomes or polycationic complexes) and physical (electroporation) transduction methods are the most common techniques for the introduction of plasmid based expression vectors into mammalian cells (15,16). Electroporation as a physical transfection method has become the preferred method for cell transduction because this method is very efficient and relatively easy to perform. Cell treatment with high intensity electric field pulses provokes the temporary pores in the membrane structure leading to a loss of its barrier function, a phenomenon called electro-permeabilization (7,17,18). These pores allow exogenous nucleic acid molecules to cross into the cytoplasm or nucleus (19). By a proper choice of the electroporation parameters (i.e. amplitude, voltage, burst duration and number of burst), these changes in the cell-membrane permeability may be either reversible that preserve cell viability or irreversible leading to cell death. In this method, the transduction of exogenous molecules into the cell is not limited by the size and nature of a vector (20,21). Therefore, the technical possibility to introduce (load) exogenous compounds like drugs and plasmids into cells is practicable by electroporation method. This technique is now used as a very efficient way for drug, oligonucleotide, plasmid, and antibody delivery *in vitro* and *in vivo* for clinical applications (17,18). 

Many studies have now shown that plasmid electro-transfer can lead to a long-lasting therapeutic effect in some diseases, such as cancer, blood disease, or muscle ischemia (22,26). There are several reports of successful transfection of different cells including heart myoblast cells (27), mammary epithelial cells (28), retinal and iris pigment epithelial cells (29), dental pulp stem cells (30), adipose and mesenchymal stem cells (31), embryonic and adult neural stem cells (32), etc., through electroporation. Since stem cells are considered to be able to propagate infinitely, *in vitro* transduction and expansion of transfected SSCs are necessary for *in vitro* development assay, fertility preservation, disease modeling, male infertility treatment, and production of transgenic animals (1,2). 

Due to the low transfection efficiency of electroporation in spite of its advantages and the importance of this germ line, considerable efforts should be performed to establish more efficient protocols for transfected SSCs line generation. The transfection efficiency of electroporation is highly dependent on the cell environment and conditions in which electric pulse are applied. In some cases, electroporation parameters utilized under one condition for transfecting a particular cell line may not necessarily be optimal for another cell line. Thus, the transfection protocol should be specifically optimized for each condition and each type of cell line. In present study, we investigated the effect of electroporation parameters including total volt, burst duration, number of bursts on total transfection efficiency, viability rate and mean fluorescence intensity (MFI) of testicular cells including SSCs. In order to improvement of the transfection efficiency and increasing the permeability of cell membrane, we used dimethyl sulfoxide (DMSO) as a transfection enhancing reagent to transduction medium and evaluated above parameters in all groups. 

## Materials and Methods

All experimental procedures were carried out with the recommendations in the guidelines for the care and use of animals by Avicenna Research Institute Animal Care and Use Committee. 

## Cell isolation and preparation

This study is an experimental research that conducted in Biotechnology Research Center (Avicenna Research Institute, Tehran, Iran) from September 2013 to March 2014. Testis samples were collected from sheep between 1 to 3 months of age at a commercial slaughterhouse and transported to the lab in transition media [phosphate buffered saline (PBS) supplemented with 100 IU/ml penicillin (GibcoBRL, USA) and 100 μg/m1 streptomycin (GibcoBRL, USA)] in an ambient temperature. Testicular cell suspensions were prepared using a protocol previously described (33). Briefly, after collection of testes and removing the tunica albuginea and visible connective tissues, the testes samples were minced with fine scissors and transferred into the Dulbeco Modified Essential Medium (DMEM, GibcoBRL, USA) supplemented with 14 mol/L NaHCO (Sigma, Germany), 10 µl/ml nonessential amino acids (NEAA, Sigma, Germany), 50 IU/ml penicillin and 50 mg/ml streptomycin for 5-8 minutes. The SSCs were isolated through two-step digestion method by collagenase type 1 (1 mg/ml, Gibco Burlington, Canada) and trypsin-EDTA (0.25%/1 mM, Sigma, Germany), respectively. The suspension was filtered successively through 60 μm nylon mesh (Small Parts Inc., Miramar, FL, USA). The filtrate was centrifuged at 500 xg for 5 minutes, and the pellet was then resuspended in DMEM supplemented with 10% fetal bovine serum (FBS, Gibco, USA). In the final cell suspension, total cell number and viability rate were determined by Trypan Blue (Sigma, Germany) staining. 

## Identification of testicular cells

Testicular cells were seeded at a concentration of 2×10^4^ cells/cm^2^ in 12-well chamber slide (Falcon, USA) at 38˚C, in a humidified atmosphere of 5% CO^2^ for 4-5 days. The basic culture medium was consisted of high glucose DMEM supplemented with 10% FBS, 10 µl/ml NEAA, and 1% penicillin-streptomycin. The spermatogonia and Sertoli cells were identified by light and fluorescence microscopes (Olympus, Japan) for detection of KIT and vimentin positive cells using immunostaining techniques. Peritubular myoid and Leydig cells were distinguished easily from the other testicular cells by a light microscope because of their specific morphological characteristics. 

## Immunocytochemical staining

The Sertoli cells were identified through vimentin immunocytochemical staining according to the protocol previously described (3). For this purpose, 2-3 days after culture initiation, the monolayer was washed with PBSTween 20 (0.2% in PBS, Sigma, USA), fixed in acetone and incubated with the primary antibody including anti-vimentin antibody (Abcam, UK) for 1 hour. After washing three times with PBS-Tween 20 (5 minutes each), the cells were exposed to secondary antibody [Fluorescein isothiocyanate (FITC)-conjugated sheep anti-mouse IgG; Avicenna Research Institute, Iran] for 45 minutes at room temperature. The nuclei were counterstained by 4,6-diamidino2-phenylindole dihydrochloride (DAPI, 1 µg/ ml, Calbiochem, UK) for 20 minutes and examined under a fluorescence microscope. SSCs were identified through KIT immunocytochem428 ical staining according to the protocol previously described (34). Briefly after washing the collected cells with PBS-Tween 20, approximately 5×10^4^ cells were centrifuge dinacytospin centrifuge at 400 rpm for 5 minutes and fixed in acetone (Merck, Germany) (2 minutes in-20˚C). Antigen retrieval was performed in citrate buffer (0.01 M, pH=6.0, Merck, Germany) for 8 minutes. All sections were exposed to 0.3% H_2_O_2_ for 15 minutes in dark to inhibit endogenous peroxidase and washed in PBSTween. The unspecific sites blocking was done with avidin/biotin (Vector Laboratories Inc., USA), and 5% sheep serum for 10 minutes. Subsequently, the slides were incubated with unconjugated primary antibodies, including rabbit anti KIT (Santa Cruz, USA) at 1:400 in PBS with 2.5% goat serum (PBS-GS, Avicenna Research Institute, Iran), for 1 hour at room temperature. After three times washing in PBSTween, the sections were exposed to secondary antibody [FITC-conjugated sheep anti-rabbit IgG; Avicenna Research Institute, Iran] for 45 minutes at room temperature. At the final step, the slides were washed with xylol and mounted by glycerol/PBS (50/50) (Sigma, Germany). 

## Optimization of transfection efficiency

Testicular cells with 75-85% confluency were refreshed with culture medium approximately 2-4 hours before transfection. Then confluent cells were trypsinized and centrifuged for 10 minutes at 1200 rpm (approximately 400 xg). Cells were resuspended in ice-cold PBS buffer without any serum, antibiotic, and Ca/Mg. Cold PBS buffer containing a correct cell concentration (1×10^6^cells/ml) were put into the electroporator cuvette (0.4 mm gap Electroporation Cuvettes, PLUS BTX^®^, USA) and 2 µg of enhanced green fluorescent plasmid (pEGFP-N1, Clontech, Japan) was added. In experimental groups containing DMSO, approximately 6.6 µl DMSO was added to cuvette and completely mixed. Then, cuvette was inserted into the shocking chamber of an electroporator machine (BTX^®^)where the requested program was installed. We optimized the transfection efficiency of testicular cells by considering of different parameters such as total voltages of 280, 320, and 350 V in burst duration of 10, 8, and 5 milliseconds, respectively, and two modes, single or double bursts. We evaluated the effect of different parameters on total transfection efficiency, cell viability, and MFI of testicular cells including SSCs. As well as, the effect of DMSO as a transfection enhancing reagent on above indexes was determined in each group. All the experiments were electroporated at room temperature. Immediately after burst, the cuvettes were placed at 4˚C for 10 minutes. 

## Propagation of transfected testicular cells

Transfected testicular cells were diluted with basic culture medium and propagated in 12-well chamber slide (Falcon, USA) at 38˚C , in a humidified atmosphere of 5% CO for 48 hours. The chamber slides were coated with gelatin (0.1%) before culturing. In the final cell suspension, total cell number and viability rate were determined by Trypan Blue staining. Forty-eight hours after electroporation, cells were checked using fluorescent microscopy (Nicon-TE300, B-2A Nicon filter, Nicon, Japan) with excitation wavelength of 450-490 nm and emission wavelength of 515 nm, magnification ×100 and ×400, and gene expression was analyzed. 

## Evaluation of * eGFP* expression

The * eGFP* positive cells (% of total cells) were detected using flow cytometry analysis at 48 hours after transfection. Cells were harvested by trypsinization and resuspended in ice-cold antibioticand ca/mgfree PBS. Approximately 30×10^4^cells/500 µl PBS were investigated by a BD FACSCalibur flow cytometer (BD Biosciences, CA) and analyzed using the FlowJo software (version 7.6.1, Cracked by Min@PKU). Cells were gated and counted according to their positivity and negativity for * eGFP* gene. Also, MFI of testicular cells was evaluated by two evaluators who were blinded to the study. Each evaluator inspected at least 10 high-powered fields for examination of quantitative expression of different testicular cells and MFI was determined with ImageJ 1.37v software (National Institutes of Mental Health, USA). 

## Statistical analysis

The results were expressed as mean ± SD. The statistical significance between the mean values was determined by one-way analysis of variance (ANOVA) analysis of variance (Student Newman Keuls Method) with SigmaPlot 12.3 statistical software. A value of P≤0.001 was defined as statistical significance. 

## Results

### Characterization of sheep testicular cells

**Fig.1:**
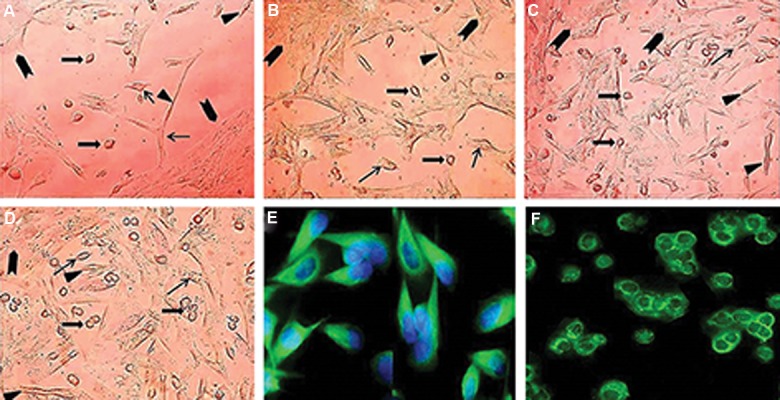
Typical morphology of somatic and spermatogonial cells during the process of cultivation at A. 1, B. 2, C. 3, D. 4 days. Somatic cells including Sertoli (arrows), Leydig (arrowheads) and myoid (triangle) cells formed the feeder layer with grown spermatogonia at its surface (block arrow), E. Immunocytochemical evaluations of sheep testicular cells after 2-3 days culture using an antibody against vimentin, and F. Spermatogonia were identified by KIT immunocytochemical staining one week after culture initiation (scale bars A-D: 50 µm, and E, F: 40 µm).

Cell recovery after tissue digestion was about 5×10^6^ cells per gram of testis. A mixed population of testicular cells with different sizes was determined in culture initiation. During the process of cultivation, the somatic cells completely attached to the culture plate after. One day after culture initiation, the morphology of adherent testicular cell were detected (Fig.1A). The Leydig cells also known as interstitial cells represent a heterogeneous cell population. These cells are light polyhedral epithelioid cells with a single eccentrically located ovoid nucleus containing one to three prominent nucleoli and large amount of peripheral heterochromatin. Their cytoplasm usually contains numerous lipid-filled vesicles (Fig.1A-D). The more abundant testicular cell types in culture condition were Sertoli cell. One of the most specific characteristics of Sertoli cells is the unique appearance of the nucleus and its tripartite nucleolus. The Sertoli cell nucleus is large and can take on several different shapes depending on the stage of the seminiferous cycle and age of development (Fig.1A-E). In testicular cell culture, the vimentin labelling was observed only in Sertoli cells (Fig.1E). Peritubular myoid cells were distinguished as a spindle fibroblastic like cells with tiny cytoplasmic rim (Fig.1A-D). After two days, somatic cells organized a monolayer with 50% confluency as a feeder layer and the most spermatogonia were attached to its surface (Fig.1B). Spermatogonia are identified as a round cell with a high nucleus: cytoplasm ratio and many cytoplasmic inclusions that were mostly concentrated at one side of the cell (Fig.1D,F). About 90% of SSCs attached to the feeder layer 2-3 days after inoculation. Four days after culture initiation, a confluent feeder layer with four types of cell was distinguished (Fig.1D). 

### Comparison of transfection efficiency

Our goal was to increase the transfection efficiency of testicular cells, especially SSCs. We increased the efficiency through modification of the electroporation parameters. The highest transfection efficiency was obtained in 320 V/8 milliseconds/single burst group in transduction medium with and without DMSO (25 ± 2.5% and 25.3 ± 2.4%, respectively, Table 1). Application of double burst for transduction of testicular cells in medium without DMSO in 280 V/10 milliseconds and 350 V/5 milliseconds groups significantly increased the transfection efficiency (P≤0.001, Table 1, Figes.2, 3). Whereas, application of both parameters, addition of DMSO to transduction medium and usage of double burst, in all voltages significantly decreased the transfection efficiency. There was a significantly inverse correlation between transfection efficiency with application of DMSO and double pulse (Table 1, Figes.2, 3). 

**Table 1:** The effect of different electroporation parameters on transfection efficiency of sheep testicular cells 48 hours after transfection


	Single burst		Double burst	
Groups	DMSO negative	DMSO positive	DMSO negative	DMSO positive

280 V/10 milliseconds	74.48 ± 1.4^A, a^	63.14 ± 0.4^B, a^	74.83 ± 0.7^A, a^	65.3 ± 2.8^B, a^
320 V/8 milliseconds	78.57 ± 1.5^A, b^	70.18 ± 1.4^B, b^	38.23 ± 0.6^C, b^	30.76 ± 2.3^D, b^
350 V/5 milliseconds	65.5 ± 1.3^A, c^	49.8 ± 1.1^B, c ^	34.7 ± 1.7^C, c ^	20 ± 2.3^D, c ^


DMSO; Dimethyl sulfoxide, ^A-D^; Numbers with different upper case superscript letters in the same column differ significantly, and ^a-d^; Numbers with different lower case superscript letters in the same row differ significantly (P<0.05).

**Fig.2:**
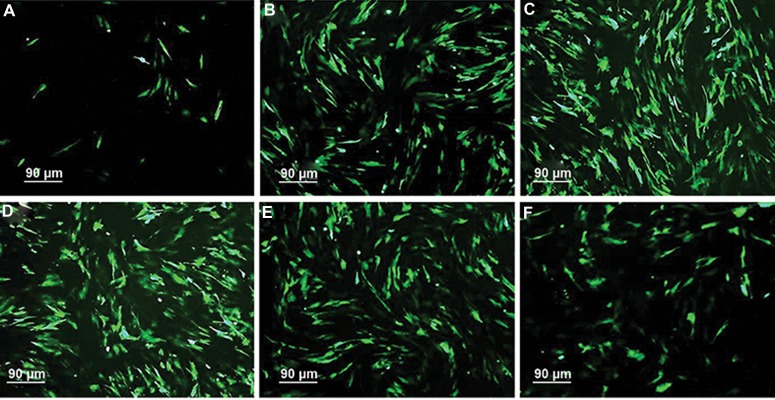
The effect of DMSO on * eGFP* expression of sheep testicular cells in A and B. 280 V/10 milliseconds, C and D. 320 V/8 milliseconds, E and F. 350 V/5 milliseconds. Testicular cells were electroporated with A., C., E. Single and B., D., F. Double bursts. Note the highest transfection efficiency 320/8 milliseconds/single burst group 48 hours after electroporation. GFP; Green fluorescent protein and DMSO; Dimethyl sulfoxide (scale bars: 90 µm).

**Fig.3:**
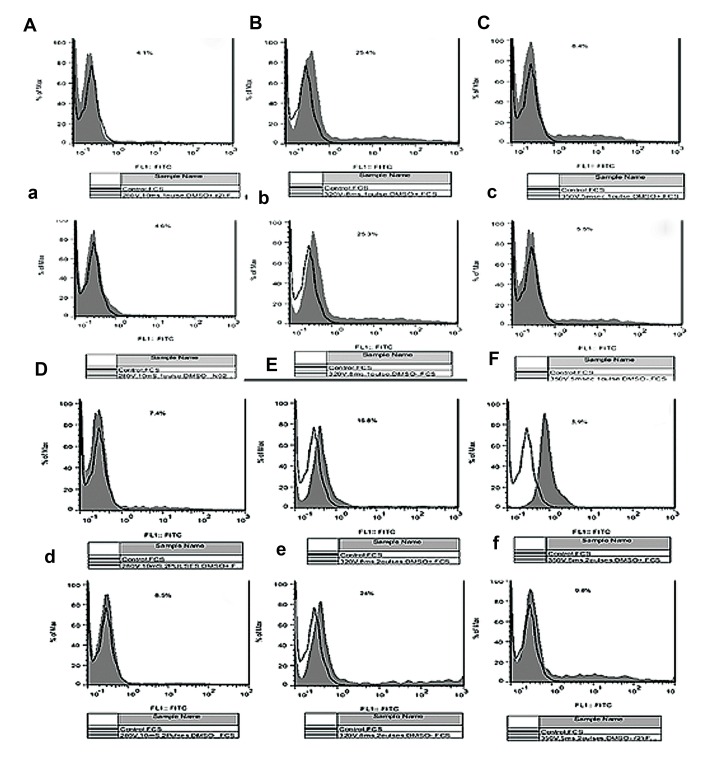
Flow cytometry histograms evaluation of transfected sheep testicular cells in experimental groups A, a, D, d. 280 V/10 milliseconds, B, b, E, e. 350 V/8 milliseconds, and C, c, F, f. 350 V/5 milliseconds with A, a, B, b, C, c. Single and D, d, E, e, F, f. Double bursts in transduc- tion medium A, B, C, D, E, F. With and a, b, c, d, e, f. Without dimethyl sulfoxide (DMSO) 48 hours after transfection.

### Effect of electroporation parameters on cell viability

Immediately after digestion and before electroporation, cell viability was observed and found to be almost identical (>95%). After transfection, the highest and lowest viability rate of testicular cells was demonstrated in 320 V/8 milliseconds with transduction medium without DMSO and 350 V/5 milliseconds in medium containing DMSO (78.57 vs. 20%, respectively, P≤0.001, Table 2). Addition of DMSO to transduction medium in all groups significantly decreased the viability rate. Moreover, application of double burst for transfection significantly destructed the testicular cells and decreased the viability rate except in 280 V/10 milliseconds group (Table 1). Therefore, if the goal was transduction of testicular cells with double burst, usage of 280 V/10 milliseconds was preferred in each group of DMSO (65.3 vs. 63.14% and 74.83 vs. 74.48%, respectively, P≤0.001, Table 2). Our data demonstrated that when the decision was transduction of testicular cells with single burst, application of 320 V/8 milliseconds would be resulted the highest transfection efficiency with maximum viable cells (Tables 1, 2). 

### Mean fluorescence intensity of testicular cells in different electroporation conditions

**Fig.4:**
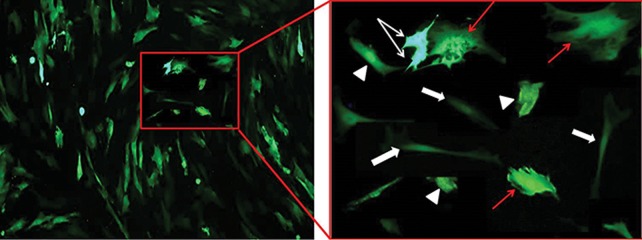
The comparison of fluorescent intensity of * eGFP* in SSCs (white arrows), Sertoli (triangles), Leydig (red arrows), and myoid (block arrows) cells after 48 hours of transfection. Note the most and least fluorescent intensity in SSCs and peritubular myoid cells, respectively, indicating amount of produced green fluorescent protein by the * eGFP* gene in these cells (scale bars: 30 µm). GFP; Green fluorescent protein, DMSO; Dimethyl sulfoxide, and SSCs; Spermatogonial stem cells.

MFI of protein produced by the reporter gene in transfected cells were demonstrated in Tables 3-5. The most and least level * eGFP* expression of testicular cells was detected in 320 V/8 milliseconds and 280 V/10 milliseconds groups, respectively (Table 1). Addition of DMSO to transduction medium significantly increased the MFI of Sertoli cells in 280 V in each burst number (49.86 ± 4.7 vs. 38.83 ± 3.4 and 47.61 ± 4.5 vs. 40.82 ± 3.4, Table 3). The spermatogonia and Leydig cells demonstrated the most fluorescence expression in medium containing DMSO in 320 V/8 milliseconds group (177.5 ± 1.6 and 80.76 ± 7, P≤0.001, Table 4). The comparison of gene expression demonstrated that Sertoli and SSCs had the most fluorescence intensity in 320 V/double burst/DMSO positive. However, myoid and Leydig cells demonstrated the most expression in 320 V/single burst and/or 350 V/double burst/DMSO positive (P≤0.001, Tables3-5). Among different testicular cell types, SSCs and peritubular myoid cells showed the highest and lowest fluorescent intensity, respectively, indicating the amount of produced green fluorescent protein by the * eGFP* gene in this cells (Fig.4). 

**Table 2:** The effect of different electroporation parameters on viability of sheep testicular cells 48 hours after transfection


	Single burst		Double burst	
Groups	DMSO negative	DMSO positive	DMSO negative	DMSO positive

280 V/10 milliseconds	74.48 ± 1.4^A, a^	63.14 ± 0.4^B, a^	74.83 ± 0.7^A, a^	65.3 ± 2.8^B, a^
320 V/8 milliseconds	78.57 ± 1.5^A, b^	70.18 ± 1.4^B, b^	38.23 ± 0.6^C, b^	30.76 ± 2.3^D, b^
350 V/5 milliseconds	65.5 ± 1.3^A, c^	49.8 ± 1.1^B, c ^	34.7 ± 1.7^C, c ^	20 ± 2.3^D, c ^


DMSO, Dimethyl sulfoxide, ^A-D^; Numbers with different upper case superscript letters in the same row differ significantly, and ^a-d^; Numbers with different lower case superscript letters in the same column differ significantly (P<0.05).

**Table 3:** Mean fluorescence intensity of sheep testicular cells in 280 V/10 milliseconds group after 48 hours of transfection


	280 V/10 milliseconds			
	Single burst		Double burst	
Testicular cells	DMSO negative	DMSO positive	DMSO negative	DMSO positive

Myoid cells	24.25 ± 1.4^a, A^	21.97 ± 2.1^a, A^	24.14 ± 1.6^a, A^	22.12 ± 2.2^a, A^
Sertoli cells	38.83 ± 3.4^a, B ^	49.86 ± 7.7^b, B^	40.82 ± 3.4^a, B^	47.6 ± 4.5^b, B^
Leydig cells	79.54 ± 7.3^a, C^	72.75 ± 6.3^b, C^	72.27 ± 6.3^b, C^	69.16 ± 4.7^b, C^
SSCs	132.5 ± 1.2^a, D^	127.66 ± 1.3^a, D ^	129.68 ± 1.1^a, D^	127.27 ± 1.3^a, D^


DMSO; Dimethyl sulfoxide, SSCs; Spermatogonial stem cells, ^A-D^; Numbers with different upper case superscript letters in the same column differ significantly, and^a, b^; Numbers with different lower case superscript letters in the same row differ significantly (P<0.05).

**Table 4:** Comparison of mean fluorescence intensity of sheep testicular cells in 320 V/8 milliseconds group after 48 hours of transfection


	320 V/10 milliseconds			
	Single burst		Double burst	
Testicular cells	DMSO negative	DMSO positive	DMSO negative	DMSO positive

Myoid cells	28.91 ± 0.01^a, A^	24.65 ± 2.4^b, A^	23.3 ± 1.9^b, A^	18.15 ± 1.6^c, A^
Sertoli cells	45.99 ± 1.9^a, B^	46.21 ± 4^a, B^	43.35 ± 3.6^a, B^	46.46 ± 4.8^a, B^
Leydig cells	75.43 ± 7.2^a, C^	80.76 ± 7^b, C^	75.54 ± 6.9^a, C^	71.23 ± 6.6^c, C^
SSCs	144.82 ± 1.4^a, D^	125.04 ± 1.1^b, D^	147.16 ± 1.3^c, D^	177.5 ± 1.6^d, D^


DMSO; Dimethyl sulfoxide, SSCs; Spermatogonial stem cells, ^A-D^; Numbers with different upper case superscript letters in the same column differ significantly, and^a, b^; Numbers with different lower case superscript letters in the same row differ significantly (P<0.05).

**Table 5:** Evaluation of mean fluorescence intensity of sheep testicular cells in 350 V/5 milliseconds group after 48 hours of transfection


	350 V/10 milliseconds			
	Single burst		Double burst	
Testicular cells	DMSO negative	DMSO positive	DMSO negative	DMSO positive

Myoid cells	22.41 ± 1.9^a, A^	23.69 ± 1.9^a, A^	20.76 ± 2^b, A^	26.28 ± 2.2^c, A^
Sertoli cells	42.63 ± 3.6^a, B^	37.83 ± 3.3^b, B^	43.16 ± 3.5^a, B ^	42.6 ± 3.7^a, B^
Leydig cells	72.72 ± 6.7^a, C^	72.3 ± 6.7^a, C^	76.19 ± 7^b, C^	79.68 ± 7.3^c, C^
SSCs	153.35 ± 1.2^a, D^	126.56 ± 1.1^a, D^	134.65 ± 1.2^a, D^	123.16 ± 1.2^a, D^


DMSO; Dimethyl sulfoxide, SSCs; Spermatogonial stem cells, ^A-D^; Numbers with different upper case superscript letters in the same column differ significantly, and^a, b^; Numbers with different lower case superscript letters in the same row differ significantly (P<0.05).

## Discussion

Production of transgenic animals has huge applications for basic science such as the creation of animal models for human diseases like Parkinson’s disease (35), as well as the production of recombinant pharmaceutics proteins in the animal’s fluid like blood, milk (36-38), egg white (11, 39, 40) and seminal plasma (41). Among the different methods of producing transgenic animals, germ cell-mediated gene transfer has great potential in development of *in vitro* assay, tissue engineering, reproductive medicine and male fertility preservation (1). One of the most commonly used cell modification methods is through transfection. Several methods were tested to acquire the most effec- tive stable transfection such as viral, physical and chemical transduction (6, 7, 12, 14). Electroporation is one of the most common and efficient non-viral transfection method used for transient expression of genes or permanent genetic modification (42).

The different investigators have indicated that due to the small pores on the cell surface made by the microseconds of cell polarization, DNA, RNA and large molecules enter the cell cytosol through simple diffusion. However, the exact mechanism is still unknown (43). Although electroporation reduces cytotoxicity, the efficiency of this method is lower than other methods. In order to apply this method in cell-based gene therapy, the transfection efficiency of electroporation should be improved. Such improvement could be achieved by modifying of electroporation parameters, such as cell densities, total voltage, burst duration, and number of bursts, and by addition of transfection enhancing reagents like DMSO (44). Our purpose was to modify the electroporation parameters and maximize the number of cells that are able to incorporate an exogenous molecule into the cytoplasm, while they are viable after transfection. For this purpose, we examined different cell densities for transduction (data not shown) and obtained the highest transfection efficiency (25.3 ± 2.4%) with appropriate viability rate (78.5%) after electroporation of 1×10^6^ testicular cells with 2 µg DNA. Our finding was consistent with the previous results indicated that the cell density of 10^5^ to 10^7^ was recommended for transfection of cock (7). Furthermore, Trefil et al. (14) demonstrated that the use of the density of 106 spermatogonial cells with 1-1.6 µg of plasmid per ml was suitable for transfection of cock SSCs. One of the most important parameter for improvement of transfection efficiency was the total voltage and burst duration, time for which the voltage was applied.

We applied the maximum times of 10 milliseconds for 280 V, 8 milliseconds for 320 V and 5 milliseconds for 350 V because a higher value of the burst duration led to cell death. The maximum number of transfected cells with more than 75% viability rate was achieved by the set of electroporation parameters: total volts (320 V), burst duration (8 milliseconds) and single burst (320 V/8 milliseconds/single burst). There was no significantly difference between treatment with or without DMSO in this condition. Kanatsu-Shinohara et al. (45) transduced mouse SSCs using single pulse 320V/200 milliseconds and achieved 20.3% positive cells with 8.7% viability rate. Furthermore, Yu et al. (10) succeeded in transfecting of cock SSCs using a single pulse of 270 V/80 milliseconds and reported the 29.37% of positive cells with 69.86% viability rate. After comparison of different condition of voltages and burst duration in transfection of chicken stem cells, Kalina et al. (7) obtained 35.8% positive cells by electroporation method using 400 V/20 milliseconds/single pulse. Cytotoxicity is critical factor that needs to be considered when assessing the safety of gene delivery methods.

In our experiments, the viability rate of testicular cells was significantly decreased after electroporation and all testicular cells including GFP-expressed cells showed the shrinkage and fragmentation of nuclei, a characteristic morphology of apoptosis. It seems that the low survival rate was due to apoptosis that indicates the electroporation process creates mini pores in cell membranes and allows small ions and molecules to pass through the membranes. This selective crossing of small ions causes osmotic swelling, which kills cells. In accordance with our result, Li et al. (6) demonstrated that DNA- uptake induced by electroporation could lead to large-scale apoptosis in human hematopoietic stem cells. After using the caspase inhibitors (B-D-Fluomethyl Ketone and Z-VAD-FMK) to reduce apoptosis, they achieved a transfection efficiency of 25% with 90% viability rate in these cells. In order to increasing the transfection efficiency, we used DMSO as an efficient penetration enhancer for gene transfer in transduction medium. It seems that DMSO improved the transfection efficiency of plasmid DNA either by increasing the permeability of cell membranes (46) and the integrity of the nucleus (47) or by affecting cell cycle synchronization (13, 48). In our experiments, addition of DMSO to transduction medium significantly increased the mean fluorescent intensity of Sertoli cells in 280 V. Our finding was consistent with the previous studies indicating that up to 8-fold increase in transfection efficiency occurred after DMSO treatment in four different cell lines (HL60, TR146, Cos-7 and L132) (49).

Villa-Diaz et al. (50) demonstrated that treatment with DMSO increased the percentage of reporter gene expressing human embryonic stem cells that was transfected by lipofection. In the other electroporation condition, we didn’t observe any improvement in the mean fluorescent signal intensity of other testicular cells using DMSO treatment. Even, addition of DMSO to transduction medium in 320 V and 280 V decreased the MFI of myoid and Leydig cells. Also, the expression of Sertoli and spermatogonia cells in transduction medium contacting DMSO was reduced in 350 V and 320 V/single pulse condition. This reduction may be due to the toxic effect of DMSO on some sheep testicular cells in specific electroporation condition. This funding was consistent with the previous result, indicating that addition of DMSO was mildly toxic to stem cells (50, 51), resulted in terminal differentiation of transformed cells (52). The contradictory effect of DMSO on different testicular cell types may be mediated through the other destructive effects on cell specific molecular mechanisms. For example, it is demonstrated that DMSO is able to inhibit c-myc expression (53), arrest cell cycle progression, and inhibit cell proliferation in different cell types (54). Furthermore, DMSO may lead to the collapse of mitochondrial membrane, release of cytochrome c from the mitochondria to cytosol, conversion of pro-apoptotic signals at the mitochondrial, and activation of caspase-9 and caspase-3 that is resulted in apoptotic changes of nuclear DNA, fragmentation of nuclei and cell death (12). Pal et al. (51) described the cellular and molecular effects of DMSO on cells such as inflammation, reactive oxygen species (ROS) scavenging, cell polarization, apoptosis, cell cycle arrest, cell differentiation, and destructive changes on molecular binding, enzyme activity, protein expression, lipid metabolism, and other experimental procedures. They also demonstrated that the higher concentration of DMSO altered the morphology and gene expression pattern of human embryonic stem cells and significantly decreased the viability rate and adhesion potential of these cells in a dose-dependent manner. They described the specific down-regulation of DMSO on some of the stemness, ectoderm, mesoderm, and endoderm molecular markers, indicating the beginning of an aberrant and untimely differentiation trajectory.

Omary et al. (55) reported the morphologic and biochemical changes in the human colonic epithelial cell line SW620 following DMSO incubation. Cells cultured in the presence of DMSO showed the striking changes by enlargement, elongation, and formation of process-like structures, using a light microscope, and a propensity to form microvillus-like structures, using an electron microscope. These changes were accompanied by significant differences in the expression of the cell surface markers CD4, CD44, and KS1, so that these changes were reversible over time upon removal of DMSO from the culture medium. Further significant variables for increasing the trans¬fection efficiency may be DNA quality, plasmid size, osmolarity and pH of transduction medium, while use of the other transfection enhancing reagents should be evaluated. Cell growth factors, cell density and log phase of the growth curve have an important influence on successful transfection of cells. The other attributes should be considered when optimization of the transformation method is undertaken. 

## Conclusion

The best combination of electroporation parameters for transfection of sheep testicular cells was recommended 320 V/8 milliseconds/single burst/ DMSO negative. The most external gene expression of Leydig and myoid cells was achieved in 350V/5 milliseconds/double pulse/DMSO positive. Whereas, the best condition for higher expression of * eGFP* in SSCs and Sertoli cells was obtained in 320 V/8 milliseconds/double bursts/ DMSO positive.
